# Use of consensus sequences for the design of high density resequencing microarrays: the influenza virus paradigm

**DOI:** 10.1186/1471-2164-11-586

**Published:** 2010-10-20

**Authors:** India Leclercq, Nicolas Berthet, Christophe Batéjat, Claudine Rousseaux, Philip Dickinson, Iain G Old, Katherine Kong, Giulia C Kennedy, Stewart T Cole, Jean-Claude Manuguerra

**Affiliations:** 1 Institut Pasteur, Laboratory for Urgent Response to Biological Threats (CIBU), Paris, France; 2 Université Paris Diderot-Paris 7, Paris, France; 3 Institut Pasteur, Genotyping of Pathogens and Public Health Technological Platform, Paris, France; 4 Institut Pasteur, Unit of Epidemiology and Pathophysiology Oncogenic Virus, CNRS URA3015, Paris, France; 5 Affymetrix, Santa Clara, CA, USA; 6 Institut Pasteur, European Office, Paris, France; 7 Institut Pasteur, Bacterial Molecular Genetics Unit, Paris, France; 8 Global Health Institute, Ecole Polytechnique Fédérale de Lausanne, Station 19, CH-1015 Lausanne, Switzerland

## Abstract

**Background:**

A resequencing microarray called PathogenID v2.0 has been developed and used to explore various strategies of sequence selection for its design. The part dedicated to influenza viruses was based on consensus sequences specific for one gene generated from global alignments of a large number of influenza virus sequences available in databanks.

**Results:**

For each HA (H1, H2, H3, H5, H7 and H9) and NA (N1, N2 and N7) molecular type chosen to be tested, 1 to 3 consensus sequences were computed and tiled on the microarray. A total of 12 influenza virus samples from different host origins (humans, pigs, horses and birds) and isolated over a period of about 50 years were used in this study. Influenza viruses were correctly identified, and in most cases with the accurate information of the time of their emergence.

**Conclusions:**

PathogenID v2.0 microarray demonstrated its ability to type and subtype influenza viruses, often to the level of viral variants, with a minimum number of tiled sequences. This validated the strategy of using consensus sequences, which do not exist in nature, for our microarray design. The versatility, rapidity and high discriminatory power of the PathogenID v2.0 microarray could prove critical to detect and identify viral genome reassortment events resulting in a novel virus with epidemic or pandemic potential and therefore assist health authorities to make efficient decisions about patient treatment and outbreak management.

## Background

Rapid detection and identification of infectious pathogens are necessary for proper treatment of infection and outbreak control measures.

Many promising approaches are being developed enabling rapid identification of various infectious pathogens such as bacteria or viruses including multiplex (RT)-PCR amplification strategies or novel technologies such as high-performance electrospray ionization mass spectrometry [1,2].

DNA microarray technologies provide a mean to screen for thousands of different nucleic acid sequences simultaneously and have been used for the detection of many viruses such as HIV [3], Hepatitis B and D viruses [4-6], human papillomaviruses [7], rotaviruses [8], vaccinia virus [9], herpesviruses, enteroviruses, flaviviruses [10], and measles virus genotypes [11]. In addition to these microarrays, which have a spectrum of identification limited to a few viral species, major microarray platforms were developed for the detection of a much broader range of pathogens [12-17].

In humans, influenza A viruses cause recurrent annual epidemics of moderate to severe respiratory diseases. Sixteen hemagglutinin (HA) molecular types (designated H1 to H16) and nine neuraminidase (NA) molecular types (designated N1 to N9) have been identified, although only a few of them occur in viruses causing human diseases. Identification and selection of sequences to be used on a microarray are particularly challenging, especially for rapidly mutating viruses. Antigenic drift is an ongoing process for influenza A viruses resulting in continuous evolution and annual epidemics. Moreover, the segmented nature of the influenza A virus genome can lead to reassortments during coinfection thus contributing to their vast genetic diversity and pandemic potential.

Subtyping and molecular analysis of influenza virus strains were performed by different groups using low density microarrays [18-23]. Most of them were used to identify influenza A viruses H1N1, H3N2, H5N1 and influenza B viruses. Recently, Combimatrix Corporation (Mukilteo, WA) developed a semiconductor-based microarray and an integrated microfluidic array enabling the detection of 15 HA and 9 NA [24,25]. However, the cost per microarray is too high for routine experimental identification. More recently, a rapid subtyping assay also identifying all the HA and NA subtypes was developed using padlock probes [2].

Resequencing, using high-density oligonucleotide microarrays, is a technology that enables the rapid identification of genetic variants and the determination of their nucleotide sequence at single base-pair resolution. This allows identification of pathogens with sequences similar but not identical to those represented on the microarray. A resequencing microarray was designed in order to allow identification of genetically diverse human RNA rhinoviruses and enteroviruses, with a minimum number of prototype 5'UTR sequences [26]. One respiratory pathogen resequencing microarray allowed the detection of geographically distant human H1N1, H3N2 and avian H5N1 influenza virus strains covering the period between 1999 and 2005 [27,28]. More recently, the same team developed a broad-range resequencing microarray for universal detection and identification of all the possible combinations of the 16 hemagglutinin and 9 neuraminidase types of avian influenza viruses in addition to three additional influenza A markers (M, PB2 and NS1) [29]. Their strategy was based on probe sequence selection using *in silico *modeling [30].

A resequencing microarray called PathogenID v2.0 has been developed by a consortium of laboratories of Institut Pasteur with Affymetrix Inc. It was used to explore various strategies of sequence selection for its design. The part dedicated to influenza viruses, presented here, was based on consensus sequences specific for one gene. Within the global program, another strategy was explored for the part of the microarray specific for rhabdoviruses. That strategy was based on the use of prototype sequences representative of each of the seven species described in the genus *Lyssavirus *and chosen within a conserved region of the polymerase gene [31]. Consensus sequences generation was *a posteriori *used to analyse results. Our strategy presented in this study, uses consensus sequences generated from nucleotide sequence alignments to determine the sequences to be tiled. These sequences characterised by a minimum size of 200 bp reduce the number of probes required for tiling yet still covering the wide diversity of influenza virus strains. To validate our approach, influenza viruses from different host origins, isolated over a period of about 50 years, were chosen. The DNA microarray not only demonstrated its ability to identify the type and subtype of influenza viruses but also had a strong discriminatory power at the level of viral variants.

## Results and Discussion

### Microarray design

The aim of the study was to detect influenza A viruses isolated over a long period of time, from a range of hosts (humans, birds, horses, and pigs) and with a minimum of sequences tiled on the array. Four genes including PB2, M, HA and NA were chosen in order to type and subtype influenza viruses. Different strategies for selecting the region to resequence were adopted depending on the particular gene. Characteristics of the sequences tiled on the microarray are detailed in Table 1. Firstly, one partially conserved sequence of 218 bp covering the majority of influenza M segments, routinely employed in medical diagnostics, was used for genus identification, namely *Influenzavirus A*, *B *and *C*. Secondly, almost one hundred sequences of PB2 genes covering a large spectrum of influenza A viruses and hosts were selected from the GenBank database. Five typical PB2 sequences representative of the diversity of PB2 sequences over a long period of time and for different host reservoirs were finally retained. The five selected sequences are described in Table 1. The strategy adopted for the hemagglutinin (HA) and the neuraminidase (NA) genes involved the design of consensus sequences for the H1, H2, H3, H5, H7 and H9 genes and the N1, N2 and N7 genes. We have restricted the number of studied molecular subtypes of HA and NA in order to make the confirmation of our hypothesis more straightforward and demonstrative without aiming to cover the whole set of serotypes. The HA and NA subtypes chosen to be tiled on the microarray are shared by all the human viruses and highly pathogenic avian viruses, and the HAs are further distributed into phylogenetic groups that gather all the 16 serotypes [32]. For each major HA and NA gene, sequences of influenza A virus were selected from different hosts including horses, pigs, humans and birds.

**Table 1:** Description of the influenza A virus sequences tiled on the PathogenID v2.0 microarray

Gene	Viral sequence identification	Position	Fragment size (bp)
M	Influenza genus identification	7..225	218

PB2	AY651706_human_H5N1_A/Dk/Indonesia/MS/2004	20..572	552
	AF258839_human_H5N1_A/Hong_kong/483/97	34..586	552
	CY005844_Avian_H5N2_A/Chicken/Chis/15224/1997	35..587	552
	CY005008_Avian_H8N4_A/mallard/Alberta/194/1992	35..587	552
	CY007810_human_H3N2_A/Canterbury/02/2005	24..576	552

HA	H1_Major_group_1	1512..1730	218
	H1_Major_group_2	1516..1741	225
	H1_Minor_group_3	1527..1720	193
	
	H2_Major_group_1	1018..1238	220
	H2_Minor_group_2	1070..1278	208
	
	H3_Major_group_1	1478..1710	232
	H3_Major_group_2	1515..1746	231
	H5_Major_1	1251..1451	200
	
	H7_Major_group_1	1496..1686	190
	H7_Major_group_1	750..940	190
	
	H9_Group_1	698..868	170
	H9_Group_2	1226..1423	197

NA	N1_Major_group_1	408..689	281
	N1_Minor_group_2	471..722	251
	N1_Minor_group_2	347..580	233
	
	N2_Group_1	246..486	240
	N2_Group_2	1211..1410	199
	N2_Group_3	459..693	234
	
	N7_Group_1	560..760	200
	N7_Group_2	871..1069	198
	N7_Group_3	1180..1378	198

Positions and sizes of the consensus HA and NA sequences were determined and selected after BLASTN analysis of the sequence.

For each gene, a global alignment including all selected sequences was performed and clusters grouping a maximum number of sequences were established. In each sequence cluster, a common region with the lowest genetic divergence was identified. A consensus sequence was automatically obtained from this common region, characterised by a minimum size of 200 bases and a divergence between the consensus sequence and any given sequence belonging to the cluster of less than 15%. Twelve HA consensus sequences and 9 NA consensus sequences were identified and their positions are detailed in Table 1. Finally, the microarray based on the resequencing approach included 27 influenza viral sequences which corresponded to 7594 bases. For each relevant base of a given consensus or prototype sequence, the array contained eight 25 mer probes (4 sense and 4 antisense). Two of the eight probes represented perfect matches, while the others corresponded to possible mismatches at the central (13^th^) position of the 25 mers. All together, 60752 probes were then tiled on the microarray, allowing subtyping of influenza viruses from different host species.

### Detection of type A influenza viruses

A total of 12 influenza virus samples were analysed by the resequencing microarray, including 2 swine, 3 human, 4 equine and 3 avian strains that originated from various locations worldwide and covered the period of time between 1956 and 2007. All samples were viral isolates, propagated either in embryonated chicken eggs or in MDCK cell cultures. They were chosen from sufficiently distant points in time to cover a large diversity of influenza viruses, especially for human and swine viruses, which were subject to major antigenic drift over the years. Avian strains were derived from cloacal samples collected from ducks in Baie de Somme, Marquenterre (France) in 2007. RNA extracts were amplified by WTA (Whole Transcriptome Amplification) and hybridised on the resequencing microarray (see Methods section). Results are summarised in Table 2. Nucleotide sequences were determined from the fluorescent signals as previously described [33] and compared with sequences in GenBank by BLASTN analysis, to identify and subtype influenza virus strains. Results for the highest hit scores were taken as strain identification. After hybridisation of WTA amplified material on the DNA microarray, all samples were positive for the presence of influenza A virus with a minimum call rate of 41% for the M gene and a call rate ranging from 35.5 to 97.4% for the PB2 gene, which is the percentage of bases determined by the resequencing algorithm. Subtypes of the different strains were identified with HA and NA sequences, giving a call rate ranging from 48.5% to 100%. Time ranges were obtained from BLASTN analysis. The sequences showing the highest alignment score were considered and duplicated sequences representing different isolates of the same strain were eliminated. The time range indicated the oldest isolate with high homology provided by the BLASTN algorithm and the most recent one. The median was calculated based upon the sequences showing the highest alignment scores (see Methods section).

**Table 2:** Resequencing microarray output for identification and characterisation of the 12 influenza A viral RNAs

Origin	Tested strain	Identification		Genes	Call rate (%)	Time range	Median
		Typing	Subtyping	Genotyping			
**Swine**			H3N2	PB2	**76.2**	1981 - 1987	1984
	A/swine/Gent/1/84 (H3N2)	A		H	**94.3**	1984	1984
				N	**92.6**	1984	1984
	
			H1N2	PB2	**60.3**	1999	1999
	A/swine/Gent/7625/99 (H1N2)	A		H	**88.8**	2000	2000
				N	**81.2**	1999	1999

**Human**			H3N2	PB2	**35.5**	1972 - 1982	1975
	A/Victoria/3/75 (H3N2)	A		H	**89.9**	1968 - 1975	1974
				N	**84.6**	1968 - 2005	1975
	
			H3N2	PB2	**67.9**	1988 - 1994	1993
	A/Johannesburg/33/1994 (H3N2)	A		H	**80.4**	1988 - 1999	1993
				N	**80.7**	1993 - 2008	2008
	
			H3N2	PB2	**93.4**	2000 - 2006	2003
	A/Wyoming/3/2003 (H3N2)	A		H	**89.9**	2002 - 2003	2003
				N	**85.8**	2001 - 2008	2006

**Equine**			H7N7	PB2	**60.7**	1956	1956
	A/equine/Prague/1/56 (H7N7)	A		H	**48.5**	1975 - 2008	2004
				N	**100**	1956 - 1992	1974
	
			H3Nx	PB2	**60.3**	1972	1972
	A/equine/Miami/1/63 (H3N8)	A		H	**65.6**	1963 - 2007	1987
				N	**-**	-	-
	
			H3Nx	PB2	**64.3**	1978-1980	1979
	A/equine/Fontainebleau/1/79 (H3N8)	A		H	**57.4**	1974 - 2008	1992
				N	**-**	-	-
	
			H3Nx	PB2	**67.3**	1976 - 1994	1989
	A/equine/Grosbois/99 (H3N8)	A		H	**69.4**	1976 - 2006	1986
				N	**-**	-	-

**Avian**			H3N2	PB2	**77.3**	2005	2005
	Viral strain 221	A		H	**84.7**	2006	2006
				N	**99.4**	2004	2004
	
			H1N1	PB2	**93**	2006	2006
	Viral strain 223	A		H	**66.5**	1976	1976
				N	**90.7**	2006 - 2007	2007
	
		A	H1N1	PB2	**97.4**	2006	2006
	Viral strain 224			H	**81.8**	2003 - 2007	2006
				N	**94.9**	2001	2001

The call rate is the percentage of bases determined by the resequencing algorithm. Time ranges indicate the oldest isolate with high homology provided by the BLASTN algorithm and the most recent one. The medians were calculated based upon the sequences showing the highest alignment scores.

For swine and human viruses, a minimum call rate of 35.5% allowed identification of influenza virus with a correct temporality. Indeed, for the majority of strains the BLASTN result was close to that of the sample strain, as outlined by median analysis. The median obtained for the NA gene from the A/Johannesburg/33/94 (H3N2) virus was relatively recent compared to the year of isolation of the strain, probably because the chosen consensus sequence for this gene was highly conserved and thus weakly discriminatory. The time range obtained for the A/Wyoming/3/2003 (H3N2) virus was narrow and limited to the year 2000, probably due to the marked antigenic drift from the year 2000 observed for the H3 gene in human influenza virus strains [34].

The epidemiology of swine influenza has become increasingly complex over the last decades. Three major influenza A virus subtypes are currently circulating in swine worldwide, but the origins and the antigenic and genetic characteristics of these swine influenza viruses differ with continents or regions of the world. A/swine/Gent/7625/99 (H1N2) virus is the result of a multiple reassortment involving a swine influenza virus with avian-influenza-like internal segments and human H1N1 and H3N2 viruses isolated in 1994 [35]. BLASTN analysis for H1N2 swine virus reflected this multiple reassortment with blasted sequences originating from human, avian and swine hosts (data not shown). Between 1983 and 1985, swine H3N2 viruses appeared in Italy. They contained avian-like internal genes and HA and NA genes from human H3N2 viruses [36]. In BLASTN analysis, the vast majority of the sequences sorted by the algorithm for glycoprotein segments were from human or swine origins only. All equine influenza virus strains were identified and subtyped with the DNA microarray, with a minimum call rate of 48.5%. Medians were often heterogeneous and far from the original strain. This is probably due to (i) the paucity of equine influenza virus sequences available in the databases, (ii) the limited genetic evolution of those viruses leading to equivalent result scores after BLASTN analysis, (iii) the fact that HA and NA sequences tiled on the microarray were shorter than PB2 sequences, due to their extreme genetic diversity in influenza A viruses. For the H3N8 viruses, recent sequences corresponded to avian strains, outlining the fact that H3N8 equine viruses were reassortants between avian viruses and equine H7N7 viruses which have not been isolated in horses for more than 20 years.

All together, the results showed that the PathogenID v2.0 DNA microarray is able not only to provide correct full subtyping of various strains from different host origins but also to identify/detect, after BLASTN, an array of strains genetically related to them. In all cases, the strains output by BLASTN belonged to the same time period thus giving a strong insight into the variant to which it is genetically, and very probably antigenically, related.

### Avian samples

Cloacal samples were collected from ducks in Baie de Somme, Marquenterre (France) in 2007 and analysed by qRT-PCR specific for the M gene. WTA amplification of viral RNA followed by hybridisation to the DNA microarray confirmed the identification of influenza viruses. Two samples were identified as H1N1 viruses and one sample as H3N2 virus. Influenza virus strain 221 was identified as H3N2 virus with a call rate of 84.7% for one of the H3 consensus sequences and 99.4% for one of the N2 consensus sequences. Influenza virus strains 223 and 224 were both identified as H1N1 virus with call rates of 90.7% and 94.9% for the N1 gene and call rates of 66.5% and 81.8% for the H1 gene, respectively (see Table 2). In order to confirm subtyping of the different viruses, primers were designed based upon resequencing sequences reconstructed by the microarray and used for RT-PCR (see additional file 1). Amplified products were cloned and analysed by classical sequencing. Sequencing results confirmed the subtyping by the DNA microarray of the 3 viral strains isolated from cloacal samples. Time ranges showed that the viral strains isolated in the environment are recent, except for the H1 gene of strain 223 (Table 2). The evolutionary rate of influenza viruses in the natural avian host is believed to be slower than in mammals, and this could explain the above result. Sequences were analysed with the BLASTN program and results with highest scores are represented in Table 3. The majority of the "classical" sequences were identical to those found after BLASTN analysis of sequences generated by the DNA microarray. The level of sequence information generated by the DNA resequencing microarray is sufficient and equivalent to that obtained by classical sequencing.

**Table 3:** Avian sequences reconstructed by the PathogenID v2.0 and BLASTN alignment

Viral strain tested	Gene	Sequence size (bp)	Subtype	BLASTN analysis of the sequence reconstructed by the DNA microarray
221	PB2	511	-	A/teal/Italy/3931-38/2005 (H5N2) *
	
	HA	209	H3	A/mallard/Finland/12072/06 (H3N8) *
	
	NA	188	N2	A/duck/Denmark/65047/04 (H5N2) *

223	PB2	516	-	A/gull/Moscow/3100/2006 (H6N2) *
	
	HA	99	H1	A/duck/Bavaria/1/77 (H1N1)
	
	NA	224	N1	A/mink/Sweden/V907/2006(H5N1) *
				A/chicken/Nigeria/1047-62/2006(H5N1)
				A/Aquatic Bird/Hong Kong/m603/98 (H11N1)

224	PB2	518	-	A/gull/Moscow/3100/2006 (H6N2) *
	
	HA	133	H1	A/goose/Italy/296426/2003 (H1N1) *
				A/duck/Italy/281904/2006(H1N1) *
				A/duck/Italy/69238/2007(H1N1) *
	
	NA	252	N1	A/Aquatic Bird/Hong Kong/m603/98 (H11N1)

Sequences corresponding to highest scores are detailed. Asterisks indicate sequences that were the same as those given by the resequencing data.

## Conclusions

The PathogenID v2.0 microarray demonstrated its ability to type and subtype influenza viruses with a minimum number of tiled sequences and to give additional information close to the individual variant level. Identification of influenza viruses was obtained within the correct period of time in most cases. However, this level of discrimination is limited by the number of sequences available in databases and their level of divergence as illustrated by results obtained with equine viruses. The PathogenID v2.0 microarray should also be able to detect a re-emerging influenza virus, that has already circulated in the population, as happened in 1977 during the Russian influenza outbreak [37].

BLASTN analysis of the sequences generated by the DNA microarray should allow the determination of the segment origin for PB2, HA and NA genes, even though the consensus sequence design was not based on the origin of the viral host. The avian H3N2 virus isolated from the environment was identified and characterised as having an H3 consensus sequence different from that obtained for human H3N2 viruses. Recently, we have tested the novel human A(H1N1) pandemic virus of swine origin, derived from multiple reassortments, with the DNA microarray and BLASTN analysis determined the segment origin for the PB2, H1 and N1 genes, which were derived from avian, classical swine virus and avian-like swine virus, respectively [38]. Our data validated the strategy of using consensus sequences with all of the serotypes tested, including H1, H3, H7 (this paper) and H5 [38] (H9 and H2 were not tested, because not available in the laboratory) and N1, N2 and N7 (this paper). The simple design strategy used in this study, based on global alignements covering the whole gene, does not need the use of a specific algorithm as the one used for the TessArray RPM-Flu resequencing microarray which was based on probe sequence selection [29]. The consensus sequences which allowed to considerably limiting the number of probes tiled on the non-specialised PathogenID v2.0 microarray would allow the determination of differential diagnosis with other respiratory viruses such as Respiratory Syncytial Virus (RSV), SARS-CoV, and also potential co-infections, which is not possible with microarrays entirely dedicated to influenza viruses. The use of WTA amplification provides considerably better sensitivity and accuracy of detection compared to random RT-PCR [14] which is compatible with the use of the PathogenID v2.0 resequencing microarray for clinical samples. Furthermore, the use of random hexamers and multiple displacement amplification allowed detection of a new virus which can not be amplified with usual specific primers as shown by the study on the last novel H1N1pdm virus responsible for 2009 influenza pandemic [38]. However, the DNA microarray failed to detect the N8 gene from H3N8 equine viruses. Here, it will be useful to tile other genes on the next generation microarray, including all HA and NA subtypes or the PB1 gene, which has often been involved in reassortment in past pandemics. In this way, one could detect as many reassortants as possible thereby impacting on public health measures.

## Methods

### Microarray design

From many available influenza virus sequences, gene-specific databases containing only the sequences of a specific gene or subtype (e.g., PB2 or H1 subtype) were created and converted to the FASTA format. Then, a multiple-sequence alignment was performed with the data set with BioNumerics software for windows (version 5.1, Applied Maths). Additionally, a neighbor-joining phylogenetic tree was created. Consensus sequences were then designed based upon global alignments of this large number of influenza virus sequences available in databanks.

### Virus samples

Influenza virus samples were all viral isolates propagated either in embryonated chicken eggs or in MDCK cell cultures. The virus type was determined by hemagglutination inhibition assay [39].

Cloacal samples collected from ducks in Baie de Somme, Marquenterre (France) were inoculated via the allantoic route on 11-day-old embryonated chicken eggs. Eggs were incubated at 35°C and allantoic fluids were harvested on day 3 postinfection. Virus titration of the fluids was performed by hemagglutination by using guinea pig erythrocytes. Influenza virus isolation was confirmed by qRT-PCR specific for the M gene.

### RNA isolation and amplification

Viral RNA from influenza samples and allantoic fluids were extracted using QIAamp Viral RNA Mini Kit (Qiagen) according to the manufacturer's instructions. Nucleic acid amplification was performed by WTA as described previously [14].

### Hybridisation to microarrays

DNA amounts obtained after amplification were quantified by Quantit (Invitrogen(r)). Five micrograms of DNA was fragmented (GeneChip(r) Resequencing Assay Kit, Affymetrix Inc.) and labelled according to the GeneChip(r) Mapping 100K Assay Manual (Affymetrix Inc.). Microarray hybridisation was conducted at 45°C and array processing was carried out according to the protocol recommended by the manufacturer (Affymetrix Inc.) as previously described [40].

### Analysis of the sequences generated by the The PathogenID v2.0 microarray

After hybridisation to the DNA microarray, the nucleotide sequences of the different genes were determined from the signals as described previously [33] and compared with sequences in GenBank by BLASTN analysis.

All sequences generated by the microarray were characterised by the call rate, which is the percentage of bases determined by the resequencing algorithm. For determining the time of emergence of the sample strain, median analysis was performed as described: the sequence generated by the microarray showing the highest call rate value was blasted against sequences available in Genbank using the BLASTN 2.2.24+ program [41]. All homologous sequences within the group of sequences showing the highest score (as categorised by the BLASTN program) were selected and their year of circulation used for median calculation and determination of the time range. If the number of these sequences was higher than 100, median analysis was performed on the 100 first sequences only. For avian influenza strains, median analysis was performed as described above and the first sequence(s) showing the highest scores were also detailed in Table 3.

### RT-PCR, cloning and sequencing

After identification and subtyping of avian influenza strains by the microarray, oligonucleotides specific for each tiled influenza virus gene were designed and used in RT-PCR assays. Five microliters of extracted RNA was amplified by SuperScript(tm) One-Step RT-PCR system with Platinum(r) *Taq *DNA polymerase (Invitrogen, Life Technologies, Carlsbad, CA). One-Step RT-PCR was performed with 25 μl reaction volume. The extracted RNA sample (5 μl) was added to RT-PCR mixture (20 μl) consisting of 1X Reaction Mix (a buffer containing 0.4 mM of each dNTP, 2.4 mM MgSO_4_), 0.5 μl of SuperScript(tm) II RT/Platinum(r) *Taq *Mix, 0.4 μM primer Forward, 0.4 μM primer Reverse, 0.2 U/μl of RNaseOUT Recombinant Ribonuclease Inhibitor and nuclease-free water. The RT and PCR were carried out with following steps: RT at 45°C for 30 min; 55°C for 15 min, 94°C for 5 min, then PCR cycle 1-30, 94°C for 30 sec, 45°C for 30 sec, 72°C for 30 sec; final extension, 72°C for 5 min.

Amplified products were extracted using QIAquick Gel Extraction kit (Qiagen) according to the manufacturer's instructions and cloned in pCR^(r)^II.1-TOPO^(r) ^TA cloning vector (Invitrogen). Sequences of influenza virus genes were obtained from 3 individual clones for each gene.

## Authors' contributions

IL and NB carried out microarray design and molecular studies. IL, NB and JCM participated in experimental design of the study and in writing the manuscript. CB and CR participated in virus culture experiments. IL, NB, PD, IGO, KK, GCK, STC and JCM participated in data analysis. All authors added corrections and suggestions to the manuscript. JCM conceived and coordinated the study.

## Supplementary Material

Additional file 1 **Sequences generated by the PathogenID v2.0 resequencing microarray with the avian influenza virus strains**. Additional data file 1 is a list of sequences in FASTA format obtained after hybridisation to the PathogenID v2.0 resequencing microarray of the avian virus samples. Click here for file
