# Supplementary material for: Use of consensus sequences for the design of high density resequencing microarrays: the influenza virus paradigm
Source: BMC Genomics. 2010 Oct 20;11:586. doi: 10.1186/1471-2164-11-586 (PMC3091733; doi:10.1186/1471-2164-11-586)
Supplement: Additional file 1 — Sequences generated by the PathogenID v2.0 resequencing microarray with the avian influenza virus strains. Additional data file 1 is a list of sequences in FASTA format obtained after hybridisation to the PathogenID v2.0 resequencing microarray of the avian virus samples. [file 1471-2164-11-586-S1.DOC]

**Additionnal File 1.** Raw sequences in FASTA format obtained after hybridization to the PathogenID v2.0 resequencing microarray of the viral RNAs extracted from allantoic fluids inoculated with avian cloacal samples. PB2 (PB2), HA (Hemag) and NA (Neura) sequences are represented. Underlined sequences correspond to primers used in RT-PCR.

**Strain 221**

>flu_virus_PB2_strain_355

acgnnnngnnncnnncngcagnctnncnctcgcgagatactgacaaaaaccactgtggaccannnnnnnnnnnnnnnccnnccnnnagcagccnnncagncgnnncnngatnnnnnnnggatgaaatggatgatggcantgnnnnnnnancgnnntncnnnccnnangataatggagatgatccctgaaagaaatgagcaaggtcagactctttggagcaaaacgaatgntgctggancagacagagtnnnnnngtcacctctgcctgngnngtggtggaacagaaatggaccaacgacaagcacagtacatcngnnannagnccgncnaacctactttgaaaaggtcgannngngnaagcntnncaannntnnnnnnncgnnnngttnccgccngnctaaaatacgccgcagggttgacnnaancccgggccatgcagatctcagtgctaaagaagcgcaagatgtcatcatggaggtcgttttcccnnacgaagncggagccaggatattgacatcagagtcacagt

>flu_virus_Hemag_strain_355

nncnctnntgaccangacatatacagagatgaggcanngnncanccggtttcagatcaaagggtgccnngcngaaanctgngngcannnncngnnggntgtggatttcctttgccatatcatgctttttgctttgtgnngnntngctggggttcattatgtgggcctgccagagaggcaatattaggtgcaacatttgcatttgagnng

>flu_virus_Neura_strain_355

gcattttctctgttgaaggcaaaagctgcattaataggtgtttttatgtggagttgataagaggaaggccacaggagactagagtatggtggacctcaaacagtattgttgtgttttgtggcacttcaggtacctatggaacaggctcatggcctgatggggcgaatatcancttc

**Strain 223**

>flu_virus_PB2_strain_397

aagagatctaatgncgcannntannactcgcgagatactaacaaaaaccactgtggaccatatggccataatcaagaaatacacatcaggaaggcaagagaagaaccctgctctcagaatgaaatggatgatggcaatgaaatatccaatcacagcggncaagagnnnnnnggngnngnncgntnaannaaatgaacaagggcagacgctttggagcaagacaaatgatgctggatcggacanagtgatggtgtctcccctagctgtaacttggtggaacaggaatgggccgacaacaagtacagtccattatccaaaagtttacaaaacatactttgagaaggttgagaggttaaaacatggaaccttcggtcccgttcatttccgaaaccaagttaaaatacgccgccgagtggaggngngcngnngcnnnncnnctctcagtgcnaaagaagcacaagatgntancatggaggtcgttttcccaaatgaagtgggagctaggatattgacatcagagtcgcaattg

>flu_virus_Hemag_strain_397

ganannngncnncgnncnnngnncnnnncgnnnnccnacnncnnannnnngnacccnannnnnngncgnngngnnacagnaatcaacannnnnntaccagattttggcgatctncnncncaccnngntnnnnnnntccgntgttagtctccctgggggcaatcagtttctggatgtgctgcnntgncnnnnctnnnngcnnn

>flu_virus_Neura_strain_397

ttgtcctgtgggtgaggctnctnnnncatataactcaaggtttgagtctgttgcttggtcagcaagtgcttntntngnntcntntagttggttgacaattggaatcnnnggtccagacaatggggccgtggctgtattgaaatacaatggcataataacagacaccatcaagagttggaggaacaacatactgagaactcaagagtctgaatgtgcatgtgtaaatggttcttgctttnctgngnngnncgccgnnna

**Strain 224**

>flu_virus_PB2_strain_399

aagagatctaatgtcgcagtctngcactcgcgagatactaacaaaaaccactgtggaccatatggccataatcaagaaatacacatcaggaaggcaagagaagaaccctgctctcagaatgaaatggatgatggcaatgaaatatccaatcacagcggncaagagaataatggagatganccnngaaagaaatgaacaagggcagacgctttggagcaagacaaatgatgctggatcggacanagtgatggtgtctcccctagctgtaacttggtggancnggaatgggccgacaacaagtacagtccattatccaaaagtttacaaaacatactttgagaaggttgagaggttaaaacatggaaccttcggtcccgttcatttccgaaaccaagttaaaatacgccgccgagtggatananncnnnggccatgcagatctcagtgctaaagaagcacaagatgttatcatggaggtcgttttcccaaatgaagtgggagctaggatattgacatcagagtcgcaattg

>flu_virus_Hemag_strain_399

nnannngnngnagnnaaaananngttggngtaaaattggaatcaattgaggcnngngnntttttggcgatctactccacagtcgccngttcccngctnntggtggtctccctgggggcaatcagtttctggatgtgttctaatgnatcgngnnnatgcagaatatncnnt

>flu_virus_Neura_strain_399

ttgtcctgtgggtgaggctccttncccatataactcaaggtttgagtctgttgcttggtcagcaagtgcttgtcnncncnnnnntagttggttgacaattggaanctctggtccagacaatggggccgtggctgtattgaaatacaatggcataataacagacaccatcaagagttggaggaacaacatactgagaactcaagagtctgaatgtgcatgtgtaaatggttcttgctttactgngnngcacgacggacc
